# Ultrasound imaging in aesthetic medicine: safety and precision in injectable procedures

**DOI:** 10.1007/s40477-026-01164-6

**Published:** 2026-05-15

**Authors:** Francesca Arrigoni, Stefania Belletti, Corrado Caiazzo, Maurizio Cavallini, Andrea Cordovana, Andrea De Santis, Alessandra Ferla Lodigiani, Riccardo Lazzari, Mario Mariotti, Valentina Merenda, Marco Francesco Papagni

**Affiliations:** 1 Agorà-Italian Society of Aesthetic Medicine, Via San Francesco D’Assisi 4/A, 20122 Milan, Italy; 2 Focus Group “Ultrasound”, Agorà-Italian Society of Aesthetic Medicine, 20122 Milan, Italy; 3 SIUMB-Italian Society for Ultrasound in Medicine and Biology, 00192 Rome, Italy

**Keywords:** Complications, Fillers, Vascular mapping, Hyaluronic acid, Botulinum neurotoxin, Cosmetic medicine

## Abstract

Injectable procedures for aesthetic purposes are on the rise in recent years, and, with them, the number of related complications. Ultrasound (US) imaging is a reliable tool that can be used in conjunction with such procedures to minimize adverse outcomes. US may be used prior to the actual injection procedure to identify anatomical anomalies and for vascular mapping. US-guided injections allow to improve precision, prevent complications, reduce the injectable volume, and to treat complications. At follow-up, US imaging allows assessment of treatment success by visualizing filler placement but is also essential for diagnosing complications and carefully planning their treatment. However, a lack of standardized protocols persists, forcing practitioners to determine US use on an individual basis. This review examines the application of US before, during, and after injectable procedures, highlighting its benefits in preventing and managing complications. We focus on the literature published since 2020, thereby providing a snapshot of current clinical practice. US imaging at different stages of injectable procedures allows precise treatments, tailored to individual patients' needs, minimizes complications, enhances practitioner confidence and patient’ trust, and ultimately achieves optimal aesthetic outcomes and high patient satisfaction. Ultimately, this review highlights the need to develop standardized protocols for the implementation of US in routine clinical practice, supporting practitioners in their decision-making processes.

**Supplementary Information:**

The online version contains supplementary material available at 10.1007/s40477-026-01164-6.

## Introduction

The demand for minimally invasive cosmetic procedures, primarily targeting the face, has significantly increased in recent years, although interest in other body areas, such as hands, neck, gluteal area, and upper back is also on the rise [1–6]. Botulinum neurotoxin is the most widely used injectable for correcting excessive muscle contractions or reducing wrinkles [7]. The use of dermal fillers is rapidly growing for augmentation interventions [1]. Although several fillers are commercially available, hyaluronic acid (HA) and calcium hydroxylapatite (CaHA) are the most popular, due to their characteristics as natural compounds that are gradually reabsorbed through physiological processes and have low immunogenic potential, reducing the risk of adverse events [8].

Overall, injectable procedures are considered safe; however, undesired aesthetic outcomes and immediate or delayed complications can occur, often requiring medical or surgical interventions [9]. Due to the growing demand, higher complication rates have recently been reported [10]. Vessel integrity can be compromised, or filler material may obstruct blood flow, resulting in vascular occlusion. The severity of these complications depends on the vessels involved and can lead to serious consequences, such as visual impairment or tissue necrosis [11]. Nodules, swelling, and inflammation can also occur due to filler or toxin misplacement or spread to adjacent tissues [12].

Although practitioners’ skills are crucial for the procedure's success, individual vascular and anatomical variability increases the risk of injection-related complications, especially when treating sensitive regions with thin skin and dense vascular networks like the periocular region [13]. Consequently, implementation of visualization techniques is essential to ensure optimal outcomes and minimize the risk of complications [14].

Ultrasound (US) examination is a non-invasive technique that provides real-time, high-resolution imaging of cutaneous and subcutaneous structures, including blood vessels, as sound waves penetrate the skin and are transmitted through fluid-filled areas [15]. In particular, the high axial spatial resolution allows detailed imaging of superficial layers, without radiation and contrast medium exposure, thus providing a safer and more effective visualization tool than magnetic resonance imaging or computed tomography in cosmetic dermatology [16, 17]. In aesthetic practice, US can be performed using B-mode or color Doppler imaging. B-mode provides a two-dimensional grayscale representation of tissue morphology, structure, and anatomical relationships, whereas Doppler imaging enables vascular examination [13]. Different probes are available for different clinical applications. In aesthetic medicine, high-frequency (20–30 MHz) and ultra-high-frequency probes (e.g., 48 and 70 MHz) are commonly used to achieve high-resolution visualization of superficial structures. Consequently, high-frequency ultrasound (HFUS) and ultra-high-frequency ultrasound (UHFUS) represent optimal tools for assessing cutaneous and subcutaneous layers [18, 19]. Recently, point-of-care ultrasound (POCUS) devices, especially handheld units, have become increasingly popular in aesthetic practice. POCUS enables real-time imaging on-site, reducing diagnosis time and guiding procedures and decision-making processes more accurately. These devices represent a highly practical and affordable choice, highly accessible and easier to integrate into clinical practice [20–22]. A recent expert consensus highlighted the importance of US integration into aesthetic procedures, expanding its use beyond diagnostic applications. Indeed, pre-procedural US imaging can support practitioners in mapping vascular and anatomical structures, while US-guided injections provide real-time visualization of material placement, improving outcomes and preventing adverse events [23].

This review offers an update on US use in aesthetic medicine, emphasizing its application before, during, and after injectable procedures to enhance precision, safety, and outcomes. Specifically, considering the rising number of reports in the literature, this review synthesizes recent literature from the past five years, providing a comprehensive overview of how US techniques are applied in current clinical practice for diagnosis, vascular mapping, injection guidance, and the assessment and correction of complications.

## Methods

A literature search was performed using the following keywords: “ultrasound”, “ultrasonography”, “echography”, “sonography”, “cosmetic”, “aesthetic”, “injectable”, “injection”, “filler, “botulin”, “botox”, and combinations thereof. Articles were selected for their relevance to the main topic.

### Ultrasound applications in aesthetic medicine

The Online Resource 1 summarizes US techniques currently employed for injection procedures in aesthetic medicine, based on recent cases reported in the literature.

#### Pre-treatment ultrasound

A thorough understanding of anatomical structures is not always sufficient to prevent complications following cosmetic injections, due to individual variations [13]. Findings from Doppler US, high-resolution angiography, and cadaveric dissections proved that facial vascularity differs significantly among individuals, with arteries exhibiting varying depths, courses, and terminations [24, 25]. Physiological variations in the facial, angular, transverse facial, and supratrochlear and supraorbital arteries have been identified using color Doppler US, emphasizing the ability of this non-invasive technique to discern individual differences [26]. Consequently, a growing number of studies have reported the implementation of pre-treatment US for vascular mapping, documenting improved treatment outcomes (Online Resource 1). Naylor and Velthuis showed that pre-procedural US in patients undergoing facial filler treatments significantly reduced bruising and vascular trauma compared to blind injections [14]. Shekarriz and colleagues investigated the complex nasal vascularity, highlighting the risk of performing blind non-surgical rhinoplasty. Although blind midline deep-plane injections are traditionally considered safe, Doppler US showed that half of participants had at least one deep midline vessel, making blind injections in these subjects highly risky for vascular complications [27]. Menjívar and colleagues emphasized that surgical rhinoplasty notably affects nasal vascularization. Consequently, for post-rhinoplasty imperfections that require rhinofiller correction, pre-treatment US evaluation is crucial to assess these variations, reducing the risk of vascular complications, which is notably higher than in non-operated noses [28]. Forehead and glabellar filler injections are common; however, they carry a high risk of vascular compromise and embolism. Using Doppler HFUS, Park and colleagues identified superficial vessels along the midline of the forehead in up to half of subjects, underscoring the value of pre-injection vascular mapping [29]. As lip volumization is a widely performed procedure, the 9-point injection technique for dermal filler augmentation was recently introduced. This technique enables tailored treatments based on individual vascular patterns by using pre-treatment US to examine the depth and course of lip arteries. Subjects who received HA-filler injections with this technique reported high satisfaction and no complications [30].

Additionally, several recent studies have implemented pre-procedural US to assess body structures, as a precise anatomical knowledge of the targeted areas is essential for successful treatments (Online Resource 1). HFUS evaluations of midfacial fat compartments mobility in healthy volunteers showed that the superficial, but not the deep fat compartments, typically move in a cranial direction, supporting the deep supraperiosteal approach for filler-based procedures [31]. Yi and colleagues conducted US evaluations of the nasal area at seven anatomical points, revealing notable individual variation in the depth of subcutaneous tissues, in addition to its complex vascular system. This study underscores the importance of pre-treatment US for both vascular mapping and accurate determination of injection depth and angle for filler-based rhinoplasty [32]. Similarly, the aging-related impact on the three fatty laminae of the dorsal hand was examined using US imaging, given the growing demand for hand rejuvenation. The examinations identified the dorsal intermediate lamina as the layer most affected by fat loss. This large-scale study highlighted that US is a valuable tool for identifying subjects with a greater need for volumization and for achieving more natural outcomes [33].

Anatomical investigations are also crucial prior to Botulinum neurotoxin type A (BoNT-A) injections. For instance, paradoxical masseteric bulging can occur following injections into the masseter muscle due to individual variations of the deep inferior tendon (DIT), which can prevent an even toxin distribution. Shi and colleagues classified DIT types based on their anatomical features using US, highlighting the importance of tailored BoNT-A treatments [34]. Li and colleagues reviewed studies that implemented US imaging of the masseter, frontalis, trapezius, glabella, and gastrocnemius muscles prior to BoNT-A injections. The valuable insights into individual anatomical differences provided by pre-procedural US support practitioners in selecting optimal injection depths and needle types, improving aesthetic outcomes [35]. BoNT-A injections are also a popular treatment for shaping eyebrows and removing forehead wrinkles, targeting the line of convergence, the forehead region where the opposing movements of the frontalis muscle meet. In a recent study, HFUS assessments of frontal soft-tissues identified sex-related differences in frontalis muscle contraction, highlighting the importance of pre-treatment US to guide injection depth and prevent complications such as eyebrow ptosis [36]. A recent report by Zhang and colleagues underscored the importance of pre-procedural US. Tailored BoNT-A injections administered after US assessments of soft-tissue thickness at different jawline and neck anatomical sites resulted in high patient satisfaction and no pain [5].

Consequently, combining anatomical and vascular assessments before cosmetic treatments is recommended to enhance accuracy and avoid vascular and structural complications. Following this approach, Gonzalez and colleagues performed B-mode and color Doppler US of the lips before HA injection. Standard US assessed prior filler presence, tissue structure, and the orbicularis oris muscle position, while Doppler US examined arterial pathways and depths. Subsequent tailored HA injections based on individual lip morphology resulted in great patient satisfaction [37].

#### Ultrasound-guided injections

Real-time US-guided filler and neurotoxin injections aim to enhance precision and safety by enabling real-time monitoring of blood vessels and critical structures [13]. An increasing number of reports in the literature adopt an approach that combines pre-procedural US for vascular and anatomical assessments and US-guided injections (Online Resource 1). In a recent study, pre-treatment US of the temple region identified 17 soft-tissue layers, enabling selection of the optimal target area at the interfacial layer, between the superficial and deep temporal fascia. Subsequent US-guided HA injections using a 23G cannula resulted in significant improvements in temple hollowness, no side effects, and high patient satisfaction [38]. Similarly, combining pre-procedural high-frequency POCUS and handheld HFUS facilitated effective and safe HA injections for nasolabial folds, tailored to the individual vascular and anatomical patterns, by allowing real-time visualization and filler administration simultaneously [39]. Bravo and colleagues showed that despite the critical treated area, patients who received nasal filling with HFUS-guided HA injections using a 22G cannula following HFUS assessments reported optimal aesthetic outcomes and no complications [40].

Accurately targeting the correct depth and plane is essential to achieve the desired appearance in facial rejuvenation procedures. Therefore, HFUS during injection enables real-time assessment of material placement and treatment success. For instance, in one patient receiving HFUS-guided HA injections using a 27G needle in the left hemiface and a 22G cannula in the right hemiface, US imaging could show that only the needle, but not the cannula, reached the supraperiosteal plane in the temporal region [41]. Interestingly, in a randomized study, Wu and colleagues demonstrated that accurately targeting the site and depth using real-time imaging significantly improves outcomes by comparing blind and US-guided BoNT-A injections for glabellar wrinkles correction. Using the same neurotoxin dosage, US-guided procedures provided significantly better outcomes with immediate effect and sustained over a one-month period, compared to blind injections, which typically become effective after 48–72 h post-injection [42].

In addition to aesthetic interventions, recent literature has highlighted the importance of US guidance in correcting complications from filler-based injections. US-guided injections of hyaluronidase (Hyal), an enzyme that hydrolyzes HA molecules, have been reported to successfully dissolve HA-based fillers in a targeted manner [16, 43–45].

Importantly, precise injections under US guidance allow a reduction in the amount of injected material and the number of injections. Yi and colleagues demonstrated that concentrating BoNT-A injections in the upper half of the platysma muscle when treating neck aging reduces the necessity of injections in the lower half of the muscle, thereby lowering the risk of injection-related complications while maintaining efficacy [46]. In a retrospective study, Schelke and colleagues showed that prompt, high-dose blind Hyal “flooding” was ineffective for vascular complications after facial volumizing procedures. However, for most patients, a single low-dose Doppler US-guided Hyal injection was sufficient for full resolution [47].

Additionally, as evidenced in recent reports, US guidance has promoted the optimization of injection methods. For instance, a tailored US-guided deep HA injection was performed on a patient with class III malocclusion and otherwise recommended for maxillofacial surgery. US imaging guided appropriate equipment selection and injection points, allowing augmentation of the anterior nasal spine via the nasal tip, a procedure typically accomplished with surgical grafts [48]. Similarly, a novel “cross-injection” technique has been described by Tang and colleagues to treat drooping mouth corner, combining one vertical and a subsequent horizontal BoNT-A injection at specific points along the fan-shaped depressor anguli oris muscle under US guidance [49]. Interestingly, to overcome the challenges associated with needle visibility in the traditional in-plane and out-of-plane facial injections, Nie and colleagues developed three modified oblique-plane techniques tailored to different facial areas and tissue layers. Using these methods, US-guided Hyal procedures can be performed safely in 10 seconds [50]. Finally, the modified Munhoz-Cavallieri lavage protocol was developed to effectively treat filler-induced refractory facial sterile abscesses, implementing US guidance for abscess drainage and for targeted application of Hyal-based lavage solution [51].

#### Follow-up and complications evaluation

The most common uses of US imaging for aesthetic purposes are the assessment of treatment success and complications diagnosis (Online Resource 1).

Several reports have documented US implementation to evaluate the correct placement of injected material and the integrity of anatomical structures following procedures. Dermal thickness represents a measurable indicator of filler integration within skin layers, providing an objective evaluation of procedure success and filler integrity and placement [13]. Petrone and colleagues used HFUS imaging to assess CaHA facial injections by measuring dermal thickness before and after the procedure, observing an average improvement of over 50% in dermal thickness four months post-treatment [52]. Progressive dermal thickness increases was also assessed with US imaging over a three-month period in 30 patients receiving CaHA facial treatments [53]. Additionally, US was employed to measure skin elasticity, revealing improved skin retraction times over time. Skin elasticity is also a valuable marker of skin quality measurable with US, as it reflects the presence and integrity of dermal collagen fibers [53]. Similarly, significant improvements in dermal thickness and collagen density were documented using HFUS in patients after HA-based facial treatments [54–57]. Although only few studies have documented UHFUS use for aesthetic assessments, this technique provides significant benefits for cosmetic dermatology by enabling visualization of microscopic structures [19].

Using UFHUS, Salvia and colleagues demonstrated increased dermal thickness at the nasolabial folds in 13 patients after HA injections [58]. Interestingly, UFHUS was recently used to evaluate the integration of different HA-based fillers, enabling visualization of fillers' properties. Specifically, Restylane Lyft had clear borders and targeted integration, while Restylane Refyne could spread across the injected tissue plane, integrating with the layers in a diffused manner [59, 60]. Similarly, using HFUS, the integration ability of two HA fillers with different rheological properties was evaluated. Although both produced comparable facial lifting effects, US imaging revealed that the high-molecular-weight (MW) HA-filler with flexible cross-linking integrated more effectively into the tissue, supporting dynamic expressions better than the filler composed of both high- and low-MW HA and with higher cross-linking degree [61].

Identification of the specific filler is crucial for accurate diagnosis and adequate management of complications. By investigating patients who received cosmetic treatments, Urdiales-Gálvez and colleagues identified four US patterns. The “heterogeneous pattern” is characterized by alternating anechoic and hyperechoic regions, typically of healthy dermis and in biocompatible fillers integrated into the tissue, such as HA-based fillers. The “fine-grain snowfall pattern” is characterized by hyperechoic imaging and posterior echogenic shadows, typical of highly infiltrative fillers, like liquid injectable silicone. The “coarse-grain snowfall pattern” is defined by homogeneously distributed hyperechoic regions, with lower echogenic shadows, typical of particulate fillers with lower infiltration ability, such as CaHA. The “globular pattern” is characterized by anechoic cysts, suggesting liquid or semi-liquid presence, typical of non-resorbable materials, like polyacrylamides, and of non-integrated HA-based fillers [62]. “Mixed” patterns can also be identified, indicating the presence of different fillers, due to multiple aesthetic treatments [62, 63]. Particularly, different studies showed that HFUS represents a valuable tool to accurately localize and characterize filling material [63–66]. In a recent report, a woman presented with a growing facial tumor. HFUS allowed visualization of a capsulated, hypoechoic pseudocyst, suggesting migrated HA filler, which was then easily removed using US-guided excision. In this particular case, US recognition of the filler avoided a serious misdiagnosis and invasive diagnostic approaches, such as skin biopsy [67].

Beyond filler identification, US allows visualization of different immediate and delayed complications (Online Resource 1). For instance, US allowed the diagnosis of specific complications in patients following BoNT-A facial injections. Specifically, high-resolution US detected an inflammatory nodule, incomplete muscle contraction, and muscular thickness asymmetry. Meanwhile, the combination of B-mode and Doppler US reclassified a presumed hematoma as a pseudoaneurysm [68]. Similarly, HFUS imaging of palpable nodules after HA injections allowed identification of specific complications, including filler deposition with or without inflammation, granulomas, and fibrosis [69]. Recently, fibrous tissue formation has been reported as a delayed complication following HA injections, affecting natural facial movement and aesthetics [70]. However, systematic US-based assessment of this specific complication are still missing, representing a potential area for future investigations. Interestingly, Wortsman and colleagues used HFUS to detect subclinical inflammatory signs by identifying abnormalities in the submandibular and parotid glands structure following filler injections. This study underscores the importance of consistently implementing post-procedural US imaging, even without evident adverse events, to preemptively identify potential inflammatory complications, preventing more serious consequences [71].

US assessment of specific complications enables targeted treatment selection, timely intervention, and reduces unnecessary invasive procedures. In a patient complaining of facial edema months after receiving poly-D-L-lactic acid filler injections, US imaging revealed multiple nodules, facilitating immediate corticosteroid therapy initiation, resulting in full resolution [72]. Choi and colleagues presented a case series in which Doppler US was used to diagnose various types of vascular compromise following HA-filler injections. The accurate recognition of these complications enabled individualized interventions, including pharmacological treatments, hyaluronidase injections, and hyperbaric oxygen therapy, highlighting the importance of early detection to prevent serious consequences [73].

Figueiredo and colleagues described a case of lip ischemia caused by vascular compression in a patient treated with HA injections. Portable HFUS and Doppler US devices were used to precisely locate affected vessels, filler material, and surrounding arteries. Consequently, targeted US-guided Hyal injections were administered, completely resolving the vascular blockage. US examinations assessed treatment success by evaluating arterial flow [74]. Similarly, several studies reported on the use of US to evaluate the effectiveness of various treatments for complications. For instance, US confirmed filler removal in patients with nodules and vascular compression caused by CaHA excess who underwent US-guided incision and filler suction [75]. US could also confirm the effectiveness of intralesional laser interventions for the resolution of HA-induced granuloma [76]. Following resolution of complications, patients may seek additional aesthetic treatments to achieve the initially desired outcomes. For this purpose, visualization techniques, such as US, are essential to ensure the complete resolution of previous complications and support a subsequent precise and safe aesthetic correction [68, 77].

Overall, post-treatment US represents an essential tool for assessing the success of aesthetic procedures and corrective interventions, and for the diagnosis of specific complications. This valuable tool can support practitioners in their decision-making process, preventing misdiagnosis and enabling prompt, targeted interventions.

## Limitations and future perspectives

Although US offers many benefits for aesthetic medicine, some challenges remain. The main limitation of US is its reliance on operator expertise. For US imaging to provide valuable information, practitioners must possess a deep knowledge of the anatomy of the treated area and of filler characteristics to achieve optimal anatomical and vascular mapping, evaluate procedural success, guide injections, and diagnose complications [13, 78]. Practitioners also need a technical understanding to select and operate the appropriate equipment for the specific task. For instance, selecting the transducer frequency and Doppler devices requires expert considerations applied to the specific function. Similarly, optimizing image quality by managing various parameters, such as depth penetration and gain, depending on the visualization purposes, is crucial to obtain clear images [79]. Additionally, when performing US-guided procedures, practitioners should manage the challenges of handling the US device while simultaneously performing injections [80]. The presence of assistants and the introduction of portable POCUS devices are important contributions to overcome these challenges, although they may present limited settings adjustability [79, 81]. Moreover, the costs of new and state-of-the-art US devices may limit their availability in various medical settings worldwide [82, 83].

To overcome these limitations, it is essential to invest in operator training and develop standardized protocols. Training of practitioners should focus on developing both practical skills, including anatomical recognition and injection techniques, and technical skills, such as US settings management and image interpretation [16, 84]. The development of clear guidelines and standardized protocols will support practitioners by reducing heterogeneity in clinical practice, improving procedure success and ensuring consistent outcomes, while providing accurate financial planning [85].

Recent advances in artificial intelligence (AI) applications for US devices and image analysis have opened new frontiers in cosmetic procedures. A recent study showed that deep learning algorithms could effectively differentiate HA and silicon oil fillers in a heterogeneous setting including different operators and various US devices worldwide. However, differentiation between CaHA and polymethylmethacrylate was not precise [86]. Although POCUS AI systems have been developed across medical specialties, limitations in their generalizability and applicability across settings persist [87]. Consequently, future research should focus on developing AI algorithms for US devices that integrate practitioners’ expertise with automated image interpretation and AI-driven surveillance systems to support the diagnosis of complications, particularly in resource-limited settings.

## Conclusions

Minimally invasive aesthetic treatments, particularly dermal fillers and BoNT-A injections, are increasingly demanded. By summarizing the latest reports from the literature, this review highlights the growing use of US for injectable aesthetic procedures. HFUS, Doppler US, and portable POCUS devices enable real-time, high-resolution visualization of cutaneous and subcutaneous structures, crucial for pre-procedural assessments, procedural guidance, and diagnosis. In particular, pre-treatment vascular and anatomical mapping is essential for identifying individual variations, preventing structural damage, and avoiding filler misplacement. US-guided injections ensure accurate material placement within targeted tissue layers, requiring less product and reducing the risk of injection-related complications. Post-treatment US assessment, the most common application reported, provides valuable information regarding procedural success and supports diagnosis of adverse events, enabling prompt, targeted management.

Consequently, US integration throughout injectable procedures allows safer, patient-tailored treatments, and enhances practitioner confidence and patient’ trust, leading to optimal aesthetic outcomes and high patient satisfaction.

Currently, only a limited number of studies have reported systematic US implementation. Therefore, the development of standardized protocols, potentially integrating AI algorithms, is necessary to consistently reduce operator bias and support practitioners in their decision-making processes.

## Supplementary Information

Below is the link to the electronic supplementary material.

Supplementary file1 (DOCX 161 KB)

## Data Availability

Data sharing not applicable to this article as no datasets were generated or analyzed during the current study.
