# Supplementary material for: Ultrasound imaging in aesthetic medicine: safety and precision in injectable procedures
Source: J Ultrasound. 2026 May 15;29(2):289–97. doi: 10.1007/s40477-026-01164-6 (PMC13250107; doi:10.1007/s40477-026-01164-6)
Supplement: Supplementary file 1 — Supplementary file1 (DOCX 161 KB) [file 40477_2026_1164_MOESM1_ESM.docx]

# Online Resource

# Journal: Journal of Ultrasound

# Ultrasound imaging in aesthetic medicine: safety and precision in injectable procedures

Francesca Arrigoni ^1^*^†^, Stefania Belletti ^2†^, Corrado Caiazzo ^3†^, Maurizio Cavallini ^1†^, Andrea Cordovana ^1†^, Andrea De Santis ^2†^, Alessandra Ferla Lodigiani ^2†^, Riccardo Lazzari ^1†^, Mario Mariotti ^1†^, Valentina Merenda ^1†^, Marco Francesco Papagni ^2†^

^1^ Agorà - Italian Society of Aesthetic Medicine, 20122 Milan, Italy

^2^ Focus Group “Ultrasound”, Agorà - Italian Society of Aesthetic Medicine, 20122 Milan, Italy

^3^ SIUMB - Italian Society for Ultrasound in Medicine and Biology, 00192 Roma, Italy

^†^ These authors contributed equally to this work and share first authorship

***** Francesca Arrigoni
Agorà - Italian Society of Aesthetic Medicine, Via San Francesco D’Assisi 4/A, 20122 Milan (MI), Italy.
Email: [f.arrigoni@agora.clinic](mailto:f.arrigoni@agora.clinic)

**Online Resource 1.** Use of ultrasound for injection procedures in aesthetic medicine, based on representative case reports and randomized controlled trials published between 2020 and 2026.

| Use of ultrasound | Injectable/procedure | References | Main purpose |
| --- | --- | --- | --- |
| Pre-treatment ultrasound | Dermal Fillers, BoNT-A, or other injectables | [1–16] | - Anatomy assessment  - Vascular mapping |
|  | -* | [17–35] |  |
| Ultrasound-guided injections | Dermal Fillers, BoNT-A, or other injectables | [3, 5, 7, 9, 13–15, 36–44] | - Precision improvement  - AEs prevention  - Injection amount reduction  - Complications management |
|  | Hyal | [45–53] |  |
| Follow-up and complication management | Success assessment | [1, 3, 4, 6, 8, 10, 12–16, 42, 43, 53–91] | - Filler placement assessment  - Vascular integrity evaluation  - Filler visualization  - Treatment success assessment |
|  | Assessment of complications from fillers | [41, 45, 47, 48, 50, 51, 53, 58, 64, 79, 87, 90, 92–116] | - Complications diagnosis  - Filler visualization  - Treatment planning |
|  | Ultrasound-guided interventions in complications management | [81, 107, 111] | - Surgical procedures targeting |

Dermal fillers include CaHA, CaHA+HA, HA, paraffin, PCL, PLLA, and RhCol-III. Other injectables include DCA, PNs, and silicone. **Abbreviations:** AEs, adverse events; BoNT-A, botulinum neurotoxin type A; CaHA, calcium hydroxylapatite; DCA, deoxycholic acid; HA, hyaluronic acid; Hyal, hyaluronidase; PCL, polycaprolactone; PLLA, poly-L-lactic acid; PNs, polynucleotides; RhCol-III, recombinant type III humanized collagen. ***:** Studies focused on anatomical and vascular mapping without subsequent injection procedure performed.

# References

1. Zhang M, Yang Y, Shi Y, et al (2025) A comprehensive ultrasound investigation of lower facial and neck structure. Aesthetic Plast Surg 49:1–12. https://doi.org/10.1007/s00266-024-04313-6

2. Naylor D, Velthuis P (2025) Integrating facial ultrasound into medical esthetics practice. J Am Acad Dermatol S0190-9622(25)02216–9. https://doi.org/10.1016/j.jaad.2025.05.1428

3. Hong G-W, Kim S-B, Koppert E, et al (2025) Filler injection techniques and ultrasound observations: ultrasound-based classification of volume deficiency in nasolabial folds. Aesth Plast Surg. https://doi.org/10.1007/s00266-025-05319-4

4. Ravera K (2025) Ultrasound and radiological patterns in assessing the efficacy of juvelook for collagen stimulation and tear trough enhancement: a case report. Cureus 17:e82668. https://doi.org/10.7759/cureus.82668

5. Lourenço LM, Di Sessa D, Tebet ACF, et al (2025) Hyaluronic high definition fill technique. J Cosmet Dermatol 24:e16692. https://doi.org/10.1111/jocd.16692

6. Gonzalez C, Callejas E, Nuñez C, et al (2025) Lip volumization with hyaluronic acid: comparative ultrasonographic evaluation of cannula and needle techniques in a multicenter study. Cureus 17:e79325. https://doi.org/10.7759/cureus.79325

7. Bravo BSF, Carvalho RDM, Elias MC, et al (2024) Nasal filling guided by high frequency ultrasound: reducing risks. J Cosmet Dermatol 23:3256–3262. https://doi.org/10.1111/jocd.16430

8. Kim JS (2024) 9-point injection technique for lip augmentation and lip corner lifting using sonographic imaging of the labial artery pathway. Aesthet Surg J 44:1080–1090. https://doi.org/10.1093/asj/sjae086

9. Shekarriz P, Shojaee P (2023) Introducing an esthetic treatment of malocclusion: ultrasound-assisted augmentation of premaxilla with injectable hyaluronic acid filler. Clin Case Rep 11:e7100. https://doi.org/10.1002/ccr3.7100

10. Wan J, Yoon SE, Rosellini I, et al (2025) Volumetric restoration and lifting of the lateral face using polycaprolactone-based filler. J Craniofac Surg 36:e564–e566. https://doi.org/10.1097/SCS.0000000000011443

11. Yi K-H, Kim S-B, Park H-J, Kim H-J (2025) Lateral eyebrow lifting and eye-opening point injection with botulinum neurotoxin: anatomical perspective. J Craniofac Surg 36:e607–e609. https://doi.org/10.1097/SCS.0000000000011112

12. Namakizadeh Esfahani N, Khorasanizadeh F, Ehsani A, et al (2025) Evaluating the efficacy and safety of deoxycholic acid injection in reduction of flank fat. J Cosmet Dermatol 24:e70436. https://doi.org/10.1111/jocd.70436

13. Kim JS, Atchima S (2025) Analysis of dermal thickness across 10 areas and injection depth after polynucleotide injection using rejumate automatic 0.8-mm 9-pin injector on the face using 33-MHz sonography. Plast Reconstr Surg Glob Open 13:e7121. https://doi.org/10.1097/GOX.0000000000007121

14. Bravo BSF, Bravo LG, Cornachini BG, et al (2025) Navigating the labial artery: a safer approach to submucosal lip filler techniques. Life (Basel) 15:509. https://doi.org/10.3390/life15040509

15. Kim J-S (2024) Efficacy, safety, and longevity of hyaluronic acid filler injection in treating temple hollowness by sonographic identifying 17 soft tissue layers. Plast Reconstr Surg Glob Open 12:e6154. https://doi.org/10.1097/GOX.0000000000006154

16. Gonzalez C, Duque-Clavijo V, Suárez S, et al (2026) High-resolution ultrasound evaluation of common dorsal hand rejuvenation techniques: a multicenter study. Cureus. https://doi.org/10.7759/cureus.101017

17. Wang L, Li T, Chi Y, et al (2023) Ultrasonographic analysis of trapezius muscle for efficient botulinum toxin type A injection. Aesthetic Plast Surg 47:2029–2036. https://doi.org/10.1007/s00266-022-03191-0

18. Park KE, Mehta P, Tran C, et al (2024) A comparison of five point-of-care ultrasound devices for use in ophthalmology and facial aesthetics. Ultrasound 32:28–35. https://doi.org/10.1177/1742271X231166895

19. Bustamante FQF, Peyneau PD, Verner FS, et al (2025) Depth and hemodynamics of the angular artery in the pyriform space: a cross-sectional study with doppler ultrasonography. Imaging Sci Dent 55:310. https://doi.org/10.5624/isd.20250075

20. Shi J, Li C, Zhou J, et al (2024) An ultrasonographic analysis of the deep inferior tendon in the masseter muscle: implications for botulinum toxin injections. Toxins (Basel) 16:391. https://doi.org/10.3390/toxins16090391

21. Meneses CCB, Freitas S, Knoedler L, et al (2024) Increasing precision during neuromodulator injections for frontal rhytids-using ultrasound imaging to identify the line of convergence. J Cosmet Dermatol 23:2373–2379. https://doi.org/10.1111/jocd.16368

22. Shekarriz P, Hosseini F, Shojaee P (2024) Ultrasound assessment of the nose vasculature: A review of the common method of non-surgical filler-based rhinoplasty. J Cosmet Dermatol 23:731–736. https://doi.org/10.1111/jocd.16037

23. Franceschelli A, Cazzulani M, Urso SU, et al (2024) In vivo ultrasound study of the angular artery anatomy: practical indications for the treatment of the deep pyriform space. Aesthet Surg J Open Forum 6:ojae113. https://doi.org/10.1093/asjof/ojae113

24. Pistoia F, Camerino PL, Ioppi A, et al (2023) High-resolution US of the facial vessels with new facial vein landmarks for reconstructive surgery and dermal injection. Eur Radiol Exp 7:49. https://doi.org/10.1186/s41747-023-00363-8

25. Khorasanizadeh F, Delazar S, Gheidari O, et al (2023) Anatomic evaluation of the normal variants of the arteries of face using color doppler ultrasonography: implications for facial aesthetic procedures. J Cosmet Dermatol 22:1844–1851. https://doi.org/10.1111/jocd.15646

26. Bravo BSF, de Melo Carvalho R, Penedo L, et al (2022) Applied anatomy of the layers and soft tissues of the forehead during minimally-invasive aesthetic procedures. J Cosmet Dermatol 21:5864–5871. https://doi.org/10.1111/jocd.15131

27. Hung Y-T, Cheng C-Y, Chen C-B, Huang Y-L (2022) Ultrasound analyses of the dorsal hands for volumetric rejuvenation. Aesthetic Surgery Journal 42:1119–1126. https://doi.org/10.1093/asj/sjac035

28. Alfertshofer MG, Frank K, Moellhoff N, et al (2022) Ultrasound anatomy of the dorsal nasal artery as it relates to liquid rhinoplasty procedures. Facial Plast Surg Clin North Am 30:135–141. https://doi.org/10.1016/j.fsc.2022.01.002

29. Schelke L, Velthuis PJ, Lowry N, et al (2021) The mobility of the superficial and deep midfacial fat compartments: an ultrasound-based investigation. J Cosmet Dermatol 20:3849–3856. https://doi.org/10.1111/jocd.14374

30. Park HJ, Lee J-H, Lee K-L, et al (2021) Ultrasonography analysis of vessels around the forehead midline. Aesthet Surg J 41:1189–1194. https://doi.org/10.1093/asj/sjaa354

31. Ten B, Kara T, Kaya Tİ, et al (2021) Evaluation of facial artery course variations and depth by Doppler ultrasonography. J Cosmet Dermatol 20:2247–2258. https://doi.org/10.1111/jocd.13838

32. Moon H-J, Lee W, Do Kim H, et al (2021) Doppler ultrasonographic anatomy of the midline nasal dorsum. Aesthetic Plast Surg 45:1178–1183. https://doi.org/10.1007/s00266-020-02025-1

33. Yi K-H, Kim S-B, Silikovich F, et al (2024) Depth of the subcutaneous tissue in the nose: application to filler injection. J Cosmet Dermatol 23:2836–2842. https://doi.org/10.1111/jocd.16346

34. Menjívar G, Heredia N, González C, et al (2024) Ultrasonographic anatomical changes in the nasal vasculature after rhinoplasty: analysis of a series of cases. J Adv Plast Surg Res 9:26–36. https://doi.org/10.31907/2414-2093.2022.09.01.3

35. Yi K-H, Kim J, Yoon SE, et al (2026) Age-related changes in the orbicularis oris muscle and their implications on aesthetic lip rejuvenation. Dermatol Surg. https://doi.org/10.1097/DSS.0000000000005035

36. Liu F, Qi G, Liu Q (2025) Advancing techniques for temporal hollowing correction: insights from ultrasound-guided PLLA injection. Aesthetic Plast Surg. https://doi.org/10.1007/s00266-025-05162-7

37. Yi K-H, Wong IKJ, Wan J (2025) Optimizing botulinum toxin injections in the platysma muscle based on motor nerve distribution. J Cosmet Dermatol 24:e70301. https://doi.org/10.1111/jocd.70301

38. Tang K, Wu Y, Wu W, et al (2025) Ultrasound-guided “cross injection” technique for mouth corner lift with botulinum toxin type A. Aesthetic Plast Surg 49:1808–1813. https://doi.org/10.1007/s00266-024-04471-7

39. Li P, Lu H (2024) Innovative approach to minimize serious complications in cosmetic dermal filling. J Vis Exp. https://doi.org/10.3791/66540

40. Wu Y, Zhang Y, Li H, et al (2024) A prospective and randomized study comparing ultrasound-guided real time injection to conventional blind injection of botulinum neurotoxin for glabellar wrinkles. J Cosmet Dermatol 23:2867–2875. https://doi.org/10.1111/jocd.16365

41. Gonzalez C, Rengifo J, Macias-Arias P, et al (2024) High-resolution ultrasound for complications of botulinum toxin use: a case series and literature review. Cureus 16:e63232. https://doi.org/10.7759/cureus.63232

42. Schelke L, Velthuis PJ, Lowry N, et al (2023) Precision in midfacial volumization using ultrasound-assisted cannula injections. Plast Reconstr Surg 152:67–74. https://doi.org/10.1097/PRS.0000000000010131

43. Oliva Hernández JL, García Monforte F, Tejero García P, et al (2022) Safety and efficacy of the transoral approach for cheek volumization with hyaluronic acid: a pilot study. J Cosmet Dermatol 21:962–969. https://doi.org/10.1111/jocd.14523

44. Bravo BSF, Elias MC, da Rocha CRM, Carvalho R de M (2021) Supraperiosteal application of hyaluronic acid with cannula: is it possible? J Cosmet Dermatol 20:3177–3180. https://doi.org/10.1111/jocd.14241

45. Lau E, Bohórquez JMC, Schelke L, et al (2025) Persistent HA fillers and nasal broadening: role of ultrasound in managing “avatar nose.” J Craniofac Surg. https://doi.org/10.1097/SCS.0000000000011283

46. Castelanich D, Parra LA, Amado AM, et al (2025) Enzymatic management of facial overfilled syndrome: a case series and narrative review. J Cosmet Dermatol 24:e70377. https://doi.org/10.1111/jocd.70377

47. Saad Y, Tannous Z (2025) Management of delayed complications of hyaluronic acid fillers: case series from the middle east. J Cosmet Dermatol 24:e70166. https://doi.org/10.1111/jocd.70166

48. Schelke L, Harris S, Cartier H, et al (2023) Treating facial overfilled syndrome with impaired facial expression-Presenting clinical experience with ultrasound imaging. J Cosmet Dermatol 22:3252–3260. https://doi.org/10.1111/jocd.16013

49. Schelke LW, Velthuis PJ, Decates T, et al (2023) Ultrasound-guided targeted vs regional flooding: a comparative study for improving the clinical outcome in soft tissue filler vascular adverse event management. Aesthet Surg J 43:86–96. https://doi.org/10.1093/asj/sjac227

50. Figueiredo HP, Coimbra F, de Carvalho Rocha T, Silva MRMAE (2024) Ultrasonography in the management of lip complications caused by hyaluronic acid. Imaging Sci Dent 54:296–302. https://doi.org/10.5624/isd.20240014

51. Cavallieri F, Munhoz G, de Almeida Balassiano LK, et al (2025) Management of hyaluronic acid filler-induced refractory sterile abscess with modified munhoz-cavallieri lavage protocol: a case series. J Cosmet Dermatol 24:e70435. https://doi.org/10.1111/jocd.70435

52. Nie B, Hu Z, Zhang W, et al (2025) Freehand oblique plane technique for ultrasound-guided hyaluronidase injections: a modified facial interventional method. Plastic and Reconstructive Surgery - Global Open 13:e7266. https://doi.org/10.1097/GOX.0000000000007266

53. Azizi N, Tootoonchi N, Khorasanizadeh F, et al (2025) Ultrasound-guided hyaluronidase injections for the management of filler-induced arterial ischemia: a pictorial case series and systematic review of literature. Aesthetic Surgery Journal Open Forum 7:ojaf125. https://doi.org/10.1093/asjof/ojaf125

54. Faria GEDL, Fakih-Gomez N, Tartare A, et al (2024) Hand rejuvenation with customizable hybrid fillers: premixed calcium hydroxyapatite and hyaluronic acid. Aesth Plast Surg. https://doi.org/10.1007/s00266-024-04145-4

55. Ducati EPJ, Magacho‐Vieira FN, Câmara CBF, et al (2025) Reviewing the ultrasound anatomy of the gluteal region and the mapping of fillers. J of Cosmetic Dermatology 24:e70499. https://doi.org/10.1111/jocd.70499

56. Luna S (2025) Bio‐revitalizing SkinGlow: assessing the efficacy of microcannula‐assisted treatment with cohesive polydensified matrix hyaluronic acid with glycerol (belotero revive) through ultrasound elastography and corneometry. J of Cosmetic Dermatology 24:e70509. https://doi.org/10.1111/jocd.70509

57. Bravo BSF, Gonçalves Bravo L, Gouvea BF, Alves GLT (2025) Leveraging anatomy to improve safety and efficacy in temple augmentations—a case series of 20 patients. Front Surg 12:1603177. https://doi.org/10.3389/fsurg.2025.1603177

58. Zhang Z, Jia Y, Tawulan T, et al (2025) Application of high-frequency ultrasound for detection and characterization of dermal fillers in the periorbital region. Plast Reconstr Surg Glob Open 13:e7067. https://doi.org/10.1097/GOX.0000000000007067

59. Lazzari R, Gallo G, Madeddu F, et al (2025) Effects of a combined collagen stimulation procedure consisting of 675 nm laser device and hyaluronic acid-based filler injections for facial rejuvenation. Photobiomodul Photomed Laser Surg 43:346–353. https://doi.org/10.1089/photob.2025.0011

60. Tao L, Yi Y (2025) Recombinant type III humanized collagen solution for injection improves skin photoaging in chinese population: a case series. J Cosmet Dermatol 24:e70276. https://doi.org/10.1111/jocd.70276

61. Bravo BSF, Bravo LG, Gouvea BF, et al (2025) Calcium hydroxylapatite-based fillers in facial rejuvenation: a prospective, single-center, unblinded comparative outcome study of Radiesse® vs. Rennova® diamond intense. J Clin Med 14:4072. https://doi.org/10.3390/jcm14124072

62. Petrone G, Leão C, Morais D, et al (2025) Clinical evaluation of an innovative CaHA bio-stimulator in facial rejuvenation: 1 year follow-up. J Cosmet Dermatol 24:e70210. https://doi.org/10.1111/jocd.70210

63. Majewska L, Dorosz K, Kijowski J (2025) Facial skin density enhancement using hyaluronic acid-based bioactive hydrogel: cannula-assisted delivery and ultrasound evaluation in a retrospective controlled study. Pharmaceutics 17:553. https://doi.org/10.3390/pharmaceutics17050553

64. Yi K-H, Wan J (2025) Compensatory masseteric bulging: a novel observation and its implications for botulinum neurotoxin injection techniques. J Cosmet Dermatol 24:e70090. https://doi.org/10.1111/jocd.70090

65. Kim J (2025) Sonographic analysis of CureJet injection depth consistency after abdominal stretch mark treatment using hyperdiluted calcium hydroxylapatite. Plast Reconstr Surg Glob Open 13:e6654. https://doi.org/10.1097/GOX.0000000000006654

66. Turcza JF, Bartosinska J, Raczkiewicz D (2025) Critical ischemia following hyaluronic acid filler injection: a case report. J Clin Med 14:802. https://doi.org/10.3390/jcm14030802

67. Samadi A, Ahmadian Yazdi H, Kafi H, et al (2025) Efficacy evaluation of a hyaluronic acid dermal filler containing mannitol: clinical and aesthetic assessment using high-frequency ultrasound. Dermatol Surg 51:46–51. https://doi.org/10.1097/DSS.0000000000004355

68. Shelemba E, Olshanska O, Benoit AG, Rumyantseva E (2025) Safety and efficacy of an injectable solution enriched with sodium hyaluronate, amino acids, and peptides in relation to superficial facial connective tissues (dermis and retinacular cutis). J Cosmet Dermatol 24:e16586. https://doi.org/10.1111/jocd.16586

69. Nikolis A, Enright KM, Cotofana S, et al (2025) Comparative trial evaluating a high- versus low-integration hyaluronic acid filler for contouring the jawline. Aesthetic Plast Surg 49:31–42. https://doi.org/10.1007/s00266-024-04347-w

70. Chang C-H, Zhao H-L (2025) Clavicular contouring with a novel subperiosteal filler injection technique: a single-case study. Aesthetic Plast Surg 49:308–313. https://doi.org/10.1007/s00266-024-04485-1

71. Weiner SF, Hicks JA, Nguyen T, Meckfessel M (2025) Split-face comparison of two hyaluronic acid fillers: intersection of rheology and tissue behavior in midface rejuvenation. Aesthet Surg J Open Forum 7:ojaf006. https://doi.org/10.1093/asjof/ojaf006

72. Zubair R, Ishii L, Loyal J, et al (2024) SPLASH: split-body randomized clinical trial of poly-l-lactic acid for adipogenesis and volumization of the hip dell. Dermatol Surg 50:1155–1162. https://doi.org/10.1097/DSS.0000000000004417

73. Guo Y, Li P, Wei W, et al (2024) Safety and efficacy of the amino acid crosslinked hyaluronic acid in the treatment of temple hollowing. Asian J Surg S1015-9584(24)02414-X. https://doi.org/10.1016/j.asjsur.2024.10.140

74. Nikolis A, Enright KM, Cotofana S, et al (2024) Intracorporeal evaluation of hyaluronic acid fillers with varied rheological properties and correlations with aesthetic outcomes. Skin Res Technol 30:e13838. https://doi.org/10.1111/srt.13838

75. Gouveia RS, Tostes LL, Bezerra FV, et al (2024) High-frequency ultrasound in the assessment before and after applying HArmonyCa^TM^. J Contemp Dent Pract 25:10–14. https://doi.org/10.5005/jp-journals-10024-3627

76. Wortsman X, Quezada N, Peñaloza O, et al (2023) Ultrasonographic patterns of calcium hydroxyapatite according to dilution and mix with hyaluronic acid. J Ultrasound Med 42:2065–2072. https://doi.org/10.1002/jum.16226

77. Salvia G, Zerbinati N, Manzo Margiotta F, et al (2023) Ultra-high-frequency ultrasound as an innovative imaging evaluation of hyaluronic acid filler in nasolabial folds. Diagnostics 13:2761. https://doi.org/10.3390/diagnostics13172761

78. Urdiales-Gálvez F, Braz A, Cavallini M (2023) Facial rejuvenation with the new hybrid filler HArmonyCa^TM^: clinical and aesthetic outcomes assessed by 2D and 3D photographs, ultrasound, and elastography. J Cosmet Dermatol 22:2186–2197. https://doi.org/10.1111/jocd.15706

79. Piccolo D, Mutlag MH, Pieri L, et al (2023) Novel management of granuloma formation secondary to dermal filler with intralesional 1444 nm Nd:YAG laser technique. Medicina (Kaunas) 59:1406. https://doi.org/10.3390/medicina59081406

80. Bravo BSF, Almeida TSCD, Carvalho RDM, et al (2023) Dermal thickness increase and aesthetic improvement with hybrid product combining hyaluronic acid and calcium hydroxyapatite: a clinical and sonographic analysis. Plastic and Reconstructive Surgery - Global Open 11:e5055. https://doi.org/10.1097/GOX.0000000000005055

81. Cohen SR, Patton S, Wesson J, et al (2023) Radiesse rescue: a preliminary study for a simple and effective technique for the removal of calcium hydroxyapatite-based fillers. Aesthet Surg J 43:365–369. https://doi.org/10.1093/asj/sjac299

82. Ehlinger-David A, Gorj M, Braccini F, et al (2023) A prospective multicenter clinical trial evaluating the efficacy and safety of a hyaluronic acid-based filler with Tri-Hyal technology in the treatment of lips and the perioral area. J Cosmet Dermatol 22:464–472. https://doi.org/10.1111/jocd.15169

83. Bezpalko L, Filipskiy A (2023) Clinical and ultrasound evaluation of skin quality after subdermal injection of two non-crosslinked hyaluronic acid-based fillers. Clin Cosmet Investig Dermatol 16:2175–2183. https://doi.org/10.2147/CCID.S402409

84. Jiang L, Yuan L, Li Z, et al (2022) High-frequency ultrasound of facial filler materials in the nasolabial groove. Aesthetic Plast Surg 46:2972–2978. https://doi.org/10.1007/s00266-022-02967-8

85. Majewska L (2022) Synergy of stabilized and nonstabilized hyaluronic acid soft tissue fillers in skin density and skin thickness enhancement. Dermatol Ther 35:e15885. https://doi.org/10.1111/dth.15885

86. Li Z, Li Z, Li Y, et al (2022) Ultrasonographic observation of the masseter muscle after injection of different botulinum toxin type A. J Cosmet Dermatol 21:5555–5561. https://doi.org/10.1111/jocd.15106

87. Munia MA, Munia CG, Parada MB, et al (2022) Doppler ultrasound in the management of vascular complications associated with hyaluronic acid dermal fillers. J Clin Aesthet Dermatol 15:40–43

88. Urdiales-Gálvez F, Barres-Caballer J, Carrasco-Sánchez S (2021) Ultrasound assessment of tissue integration of the crosslinked hyaluronic acid filler VYC-25L in facial lower-third aesthetic treatment: a prospective multicenter study. J Cosmet Dermatol 20:1439–1449. https://doi.org/10.1111/jocd.13632

89. Nikolis A, Enright KM, Öhrlund Å, et al (2021) A randomized, split-face, double-blind, comparative study of the safety and efficacy of small- and large-particle hyaluronic acid fillers for the treatment of nasolabial folds. J Cosmet Dermatol 20:1450–1458. https://doi.org/10.1111/jocd.13668

90. Trevidic P, Andre P, Benadiba L, et al (2020) Objective 18-month comparison of the tolerability of 2 dermal fillers formulated with tri-hyal technology. Plast Reconstr Surg Glob Open 8:e3274. https://doi.org/10.1097/GOX.0000000000003274

91. Moon KY, Hur JH, Yoon SE, et al (2025) Penile volume augmentation with hyaluronic acid fillers: ultrasound observation. Plastic and Reconstructive Surgery - Global Open 13:e7317. https://doi.org/10.1097/GOX.0000000000007317

92. Chen X-R, Yu Z-L, Ren J-G (2025) Managing complications in facial cosmetic treatment. J Craniofac Surg 36:1725–1730. https://doi.org/10.1097/SCS.0000000000011178

93. Tafur MG, Rodríguez-Cerdeira C (2025) Granulomatous reactions following the injection of multiple aesthetic microimplants: a complication associated with excessive filler exposure in a predisposed patient. Reports 8:194. https://doi.org/10.3390/reports8040194

94. Andrade-Hernández PK, Hernández-Chávez LA, Hernández-Alvarez AE, Vallejo-Pérez E (2025) Angioedema associated with hyaluronic acid injection. Rev Alerg Mex 72:75. https://doi.org/10.29262/ram.v72i3.1511

95. Lauria J, Zappalà G, Sidoti FC, et al (2025) Paraffinoma of the penis following subcutaneous paraffin injections: a case report and surgical management. Int J Impot Res. https://doi.org/10.1038/s41443-025-01169-5

96. Wortsman X, Valderrama Y, Ortiz-Orellana G, et al (2025) International multicentric study on ultrasound characteristics, layer location, and corporal distribution of granulomas after cosmetic fillers injections. J Ultrasound Med 44:1447–1455. https://doi.org/10.1002/jum.16700

97. Jiang L, Chen F, He M, et al (2025) Treatment of delayed hypersensitivity after injection of nasal fillers. J Cosmet Dermatol 24:e16681. https://doi.org/10.1111/jocd.16681

98. Hawkins M (2025) Bilateral submental superficial thrombophlebitis following hyaluronic acid filler injections. J Clin Aesthet Dermatol 18:S38–S39

99. Castelanich D, Parra LA, Rodriguez Cabrales JS, et al (2025) Toxin‐induced nodules: a clinically distinct complication with implications for aesthetic practice. Case Reports in Dermatological Medicine 2025:9921924. https://doi.org/10.1155/crdm/9921924

100. de Melo M da GB, Azevedo LH, Ruiz LFN, et al (2024) Photobiomodulation therapy in the management of late complications after facial filling. Cureus 16:e59513. https://doi.org/10.7759/cureus.59513

101. Yi K-H, Bae H, Kim S-B, et al (2024) Sonographic observation of the paradoxical masseteric bulging and clinical implication of functional compartment. Anat Cell Biol 57:13–17. https://doi.org/10.5115/acb.23.234

102. Pascali M, Chirico F, Rugge L, Rauso R (2024) Aesthetic surgical pathway in permanent facial filler removal. Facial Plast Surg 40:19–30. https://doi.org/10.1055/a-2019-5131

103. Koo H-J, Hu H, Kim W, et al (2024) Do repetitive botulinum neurotoxin injections induce muscle fibrosis? Sonographic observation of the masseter muscle. J Cosmet Dermatol 23:434–440. https://doi.org/10.1111/jocd.16022

104. Perez Willis KM, Ramirez Galvez R (2024) Granuloma after the injection of poly-D,L-lactic acid (PDLLA) treated with triamcinolone. Case Rep Dermatol Med 2024:6544506. https://doi.org/10.1155/2024/6544506

105. Choi SY, Shin SH, Seok J, et al (2023) Management strategies for vascular complications in hyaluronic acid filler injections: a case series analysis. J Cosmet Dermatol 22:3261–3267. https://doi.org/10.1111/jocd.15990

106. Xiong C, Xu H, Yang Z, et al (2023) Complications following facial injection of growth factor solution. Aesthetic Plast Surg 47:612–621. https://doi.org/10.1007/s00266-022-03033-z

107. Magacho-Vieira FN, Santana AP (2023) Displacement of hyaluronic acid dermal filler mimicking a cutaneous tumor: a case report. Clin Cosmet Investig Dermatol 16:197–201. https://doi.org/10.2147/CCID.S398014

108. Carella S, Ruggeri G, La Russa R, et al (2022) Clinical management of complications following filler injection. Aesthetic Plast Surg 46:886–894. https://doi.org/10.1007/s00266-021-02650-4

109. Wortsman X, Moll-Manzur C, Ramírez-Cornejo C, et al (2021) Ultrasonographic subclinical signs of inflammation of the lacrimal, parotid, and submandibular glands in users of cosmetic fillers. J Ultrasound Med 40:2377–2389. https://doi.org/10.1002/jum.15621

110. Suchyta MA, Hunt CH, Eiken P, Mardini S (2021) Intraoperative ultrasound imaging in silicone filler removal. J Craniofac Surg 32:e276–e278. https://doi.org/10.1097/SCS.0000000000007196

111. Bravo BSF, de Melo Carvalho R, Souza E, et al (2021) Ultrasound-guided poly-l-lactic acid nodule excision: The importance of the injector ultrasonographic experience. J Cosmet Dermatol 20:417–419. https://doi.org/10.1111/jocd.13527

112. Bondarenko I, Privalova E, Shumina Y (2021) Sonography of the face and neck region soft tissues in assessment of the complications causes after facial contouring. Georgian Med News 74–79

113. Mlosek RK, Migda B, Skrzypek E, et al (2021) The use of high-frequency ultrasonography for the diagnosis of palpable nodules after the administration of dermal fillers. J Ultrason 20:e248–e253. https://doi.org/10.15557/JoU.2020.0044

114. Jaguś D, Skrzypek E, Migda B, et al (2021) Usefulness of Doppler sonography in aesthetic medicine. J Ultrason 20:e268–e272. https://doi.org/10.15557/JoU.2020.0047

115. Kim JSTW, Dos Santos Guadanhim LR, De Barros Nunes GJ, et al (2020) Herpes zoster as a differential diagnosis for ischemia after facial hyaluronic acid filler. J Clin Aesthet Dermatol 13:29–31

116. Chiang J, Liao Y-H (2025) Mapping filler nodules: ultrasound characteristics and clinical patterns in dermal filler complications. Dermatol Surg. https://doi.org/10.1097/DSS.0000000000004963
